# When biomedical discovery faces data barriers: building a governance-empowered framework for resilient collaboration

**DOI:** 10.1038/s44320-025-00138-w

**Published:** 2025-08-26

**Authors:** Zefeng Wang, Guoqing Zhang, Guoping Zhao

**Affiliations:** 1 School of Life Science, Guangming Advanced Research Institute, Southern University of Science and Technology, Shenzhen, China; 2 Bio-Med Big Data Center, Shanghai Institute of Nutrition and Health, University of Chinese Academy of Sciences, Chinese Academy of Sciences, Shanghai, China; 3 Key Laboratory of Systems Health Science of Zhejiang Province, School of Life Science, Hangzhou Institute for Advanced Study, University of Chinese Academy of Sciences, Hangzhou, China

**Keywords:** Computational Biology

## Abstract

In this Comment, the authors highlight how recent shifts in U.S. data policy threaten the collaborative ethos of biomedical research and emphasize the urgent need for governance frameworks that support secure, transparent, and interoperable data sharing.

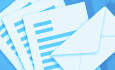

## Teaser

From the Human Genome Project to large-scale initiatives like the UK Biobank and The Cancer Genome Atlas (TCGA), the recent history of biomedical research has proven that open data sharing is a powerful engine to drive new discovery. The Human Genome Project, in particular, set a new standard by releasing genomic data publicly within 24 h (Toronto International Data Release Workshop Authors, 2009), catalyzing breakthroughs that continue to shape medicine today. This culture of openness laid the foundation for global collaboration and democratized access to scientific innovation.

Yet this collaborative model is now under serious challenge. Recent U.S. policy shifts—such as the restriction on sharing certain datasets of the National Center for Biotechnology Information (NCBI) with Chinese institutions—signal a change in international data-sharing norms. While such policies may aim to safeguard sensitive information, they also threaten to fragment the global research landscape, especially in fields like genomics and epidemiology that rely on cross-border data exchange. For researchers, this creates not only logistical challenges but also undermines the core ethos of science as a collective, transnational endeavor. These developments underscore the urgent need for governance frameworks that enable secure, transparent, and interoperable data collaboration without defaulting to isolationism.

## Data governance at the frontier of artificial intelligence (AI)-driven science

The life science of this century has entered an era where new discoveries are no longer defined solely by laboratory breakthroughs, but increasingly by our capacity to analyze and manage massive, complex datasets. The integration of AI and big data is ushering in a new “data-intensive research paradigm” (Hey et al, 2009), transforming the pace and potential of discovery. But as recent progress has shown, the true bottleneck is not in AI models themselves—it lies in data governance.

Take the case of AlphaFold 3 (Abramson et al, 2024). Its landmark advance in protein structure prediction (achieving RMSD < 1.0 Å) did not emerge from model architecture alone. It was made possible by deep, careful governance of protein databases—filtering based on crystal resolution, aligning metadata, and removing redundant non-homologous sequences. Similarly, Evo 2 model (Brixi et al, 2025) relies on the construction of OpenGenome2 system, which aggregates reference genomes from multiple global databases (including genome datasets from GTDB, IMG, NCBI, JGI, IMG, MGnify, etc.) and applies precise data preprocessing in its pretraining pipeline. These are not just technical footnotes; instead, they are the foundation of performance. These cases indicated that data governance has become the hidden engine of intelligent science, which sets the limits of what AI models can achieve in research.

Data governance in life science research refers to a structured and actionable framework that spans the entire data lifecycle—from sample collection and metadata annotation to data processing and sharing. It involves the coordinated development of data standards, technical protocols, and institutional policies that collectively ensure data quality, traceability, and reusability. At the technical level, this includes harmonized metadata formats, stable and reproducible analysis pipelines, and quality control procedures that are operational, objective, and applicable across research teams. Importantly, such procedures must go beyond file format validation to address the integrity of data content and intermediate processing steps. For instance, in transcriptome studies, quality control should assess not only FASTQ file integrity but also RNA integrity metrics (e.g., RIN scores), library complexity, and batch effects arising during alignment and quantification.

To reduce subjectivity and promote consensus, these procedures should be embedded in shared workflows and supported by transparent documentation. Traceable metadata systems—tracking parameters such as sequencing depth, sample origin, and experimental protocols—further enhance comparability and integration across datasets. At the organizational level, governance also includes access control, data versioning, authorship attribution, and ethical oversight. In this way, data governance provides the infrastructure needed to support reproducible, high-quality science under the data-intensive research paradigm.

## Knowledge-driven governance: more than just access

Yet, simply opening data is not enough. Availability does not mean usability. Raw datasets often lack the structure, quality, or consistency needed for machine learning. Only knowledge-driven, dynamic governance can turn data into actionable intelligence. For example, when the Prov-GigaPath system (Xu et al, 2024) integrated pathology images from the Providence Health Network, it faced major challenges in image variability due to device differences. Through resolution standardization, tissue segmentation, and background filtering, these inconsistencies were minimized—demonstrating the value of domain-specific governance. As cross-institutional and even cross-border data collaborations become more common, unified governance frameworks are essential. In this context, data governance acts as a technological translator, enabling collaboration beyond geographic or institutional boundaries.

The successes of large-scale open science initiatives underscore this point. The UK Biobank (Sudlow et al, 2015), for instance, not only shares existing datasets but openly communicates forthcoming resources—enabling broad and diverse health research. Similarly, MGnify (Richardson et al, 2023) provides publicly accessible tools and versioned analysis pipelines for metagenomic datasets, making it easier for researchers to apply the data effectively. These examples show that the power of data sharing lies in the governance that accompanies it. Standards, transparency, and community-driven design are what transform data into a sustainable research asset.

## Resilient governance in politically constrained settings

However, one of the most fundamental challenges in global data sharing is not technical, but political: when access to primary data is restricted or obstructed by government policy, institutional silos, or international tensions, data governance faces a critical test. In such cases, governance cannot rely solely on centralized access or unrestricted availability. Instead, it must adopt a more resilient, federated model—one that enables collaboration through shared standards, distributed control, and protocol-level interoperability, even when data cannot cross borders.

The coronavirus disease 2019 (COVID-19) pandemic exposed this dilemma at scale. While the scientific community urgently needed access to viral genomic sequences, clinical metadata, and patient outcomes from across the globe, many countries enforced strict access controls on patient-level data, citing national security, privacy concerns, or data sovereignty. In parallel, Europe’s General Data Protection Regulation posed legal constraints on cross-border sharing of identifiable biomedical data—even for public health emergencies. In such a context, federated data governance became a viable alternative: researchers were able to run analytic pipelines locally within national jurisdictions and share harmonized results or summary statistics instead of raw data. Projects such as COVID-19 Host Genetics Initiative (The C-HGI, 2020) and European Genome-phenome Archive (Freeberg et al, 2022) exemplify how shared ontologies, controlled vocabularies, and metadata alignment can facilitate international research under legal and political constraints.

These experiences emphasize that governance is not merely about managing access—it is about enabling responsible use under limitations. Embedding such governance protocols into the design of research platforms—rather than treating them as post hoc compliance layers—ensures operational continuity even when access routes are disrupted. In this sense, knowledge-driven governance serves as both a scientific enabler and a structural buffer, maintaining collaboration, integrity, and resilience in politically fragmented data environments.

## Reframing the future: build a governance-empowered scientific community

To move forward, we need to go beyond the binary of open versus closed data. The core challenge is not merely the data availability, but the usability and trust. In the age of AI and data-intensive research, raw datasets—however abundant—are not inherently valuable unless they are curated, standardized, and made interoperable. The hidden engine behind AI breakthroughs like AlphaFold 3 is not just algorithmic design, but sophisticated data governance: crystal resolution filtering, metadata harmonization, and removal of redundant sequences. These processes exemplify how intelligent science now hinges on intelligent infrastructure.

We propose a governance-empowered model of collaboration—one that treats governance not as a constraint, but as a foundational layer for scalable, ethical, and high-impact research. In this vision, governance is not simply about compliance, but about creating a common language for data across disciplines and borders. It allows research institutions to share not only data but also the standards, protocols, and quality controls that ensure its responsible use.

Four priorities stand out for building such a resilient scientific community:

- **Open governance frameworks, not just data**—Sharing protocols of data cleaning, curation, and quality assessment is often more impactful than providing unrestricted access to raw data. When developed through coordinated efforts by research institutions and consortia, such frameworks enhance methodological transparency, promote data quality, and reduce variability across studies, particularly in large-scale, multi-site investigations.
- **Design for interoperability**—Establishing governance mechanisms that accommodate diverse legal and regulatory frameworks enables data reuse and integration across institutional and national boundaries. By embedding compliance logic within data platforms and aligning metadata standards, researchers can collaborate effectively under differing policy regimes while maintaining ethical and legal integrity.
- **Quantify and reward contribution**—Using governance metadata to create traceable, reciprocal value between data contributors and users. Integrating governance metadata into research workflows allows for systematic recognition of contributions to data stewardship, pipeline development, and quality control. This approach supports a more equitable scientific ecosystem, where infrastructure work and collaborative inputs are valued alongside traditional scholarly outputs.
- **Translate governance into technical assets**—Treating governance protocols as reusable, portable infrastructure that can be integrated into data platforms and collaborative tools transforms governance into an enabler of routine scientific practice rather than a peripheral concern. When embedded within analytical pipelines, metadata models, and user interfaces, governance becomes an integral component of the research infrastructure.

By embedding these principles into the foundation of our scientific systems, we hope to turn today’s fragmented data silos into interconnected nodes of a global knowledge network. Governance, in this sense, becomes the architecture of collaboration. It provides the structural resilience needed to navigate geopolitical uncertainty while upholding the scientific tradition and necessity to share, connect, and discover. If governance is implemented effectively, we will unlock the full potential of AI for life sciences—not for any single institution or nation, but for the advancement of humanity as a whole.
